# How does the ball influence the performance of change of direction and sprint tests in para-footballers with brain impairments? Implications for evidence-based classification in CP-Football

**DOI:** 10.1371/journal.pone.0187237

**Published:** 2017-11-03

**Authors:** Raúl Reina, José Manuel Sarabia, Carla Caballero, Javier Yanci

**Affiliations:** 1 Sports Research Centre, Miguel Hernández University, Elche, Spain; 2 Faculty of Education and Sport, University of the Basque Country, UPV/EHU, Vitoria-Gasteiz, Spain; Sao Paulo State University, BRAZIL

## Abstract

The aims of this study were: i) to analyze the reliability and validity of three tests that require sprinting (10 m, 25 m, 40 m), accelerations/decelerations (Stop and Go Test) and change of direction (Illinois Agility Test), with and without ball, in para-footballers with neurological impairments, and ii) to compare the performance in the tests when ball dribbling is required and to explore the practical implications for evidence-based classification in cerebral palsy (CP)-Football. Eighty-two international para-footballers (25.2 ± 6.8 years; 68.7 ± 8.3 kg; 175.3 ± 7.4 cm; 22.5 ± 2.7 kg·m^-2^), classified according to the International Federation of Cerebral Palsy Football (IFCPF) Classification Rules (classes FT5-FT8), participated in the study. A group of 31 players without CP was also included in the study as a control group. The para-footballers showed good reliability scores in all tests, with and without ball (ICC = 0.53–0.95, SEM = 2.5–9.8%). Nevertheless, the inclusion of the ball influenced testing reproducibility. The low or moderate relationships shown among sprint, acceleration/deceleration and change of direction tests with and without ball also evidenced that they measure different capabilities. Significant differences and large effect sizes (0.53 < ηp2 < 0.97; p < 0.05) were found when para-footballers performed the tests with and without dribbling the ball. Players with moderate neurological impairments (i.e. FT5, FT6, and FT7) had higher coefficients of variation in the trial requiring ball dribbling. For all the tests, we also obtained between-group (FT5-FT8) statistical and large practical differences (ηp2 = 0.35–0.62, large; p < 0.01). The proposed sprint, acceleration/deceleration and change of direction tests with and without ball may be applicable for classification purposes, that is, evaluation of activity limitation from neurological impairments, or decision-making between current CP-Football classes.

## Introduction

Football is a multi-faceted sport, in which conditional, technical and tactical factors interact [1], requiring intermittent high-intensity efforts [2] where players are often required to repeatedly produce maximal or near maximal sprints of short duration (1–7 s) with brief recovery periods [3]. The ability of soccer players to continually perform high-intensity high-speed actions is known to impact football match performance, defined as actions requiring rapid accelerations (10 m sprint), actions at maximum speed (30 m sprint), or actions requiring agility [4]. Sheppard and Young [5] defined agility as a rapid whole-body movement with change of velocity or direction in response to a stimulus, but this definition comprises both a perceptual decision-making process and the outcome of this process, a change of direction (COD) or velocity [6]. COD can be described as the ability to change direction while sprinting over a pre-planned course [6], so linear and change-of-direction speeds are essential qualities for athletes who play field sports such as football [7, 8]. Physical match analysis showed that during elite-level football games, players perform a considerable amount of COD bouts at high intensity using a wide range of turning angles [9], and straight sprinting speed has been also considered as a factor in the football-specific component model of COD [10].

Cerebral palsy (CP)-Football is a para-sport for athletes with brain impairments such as ataxia, hypertonia or athetosis (i.e. cerebral palsy, stroke or traumatic brain injury), and those impairments that might be deemed severe enough to impact on the performance of football skills. CP-Football is currently governed by the International Federation of Cerebral Palsy Football (IFCPF), with international tournaments organized by IFCPF (i.e. Continental championships, World tournaments and U-19 championships) and by the International Paralympic Committee (IPC) or its Regional Organizations (i.e. Paralympic Games, ParaPanAm Games, Asian Paragames, or European Paragames). Considering this high international repercussion, CP footballers deserve special attention. For a particular competition, players are classified in one of the following four profiles [11]: i) FT5 athletes with hypertonia or spasticity in both lower limbs and to some degree in both upper limbs. These players have difficulty running, turning and stopping because of a lack of lower limb control; ii) FT6 athletes are affected by coordination and balance problems in all four limbs and trunk, and typically have difficulties dribbling the ball when running, accelerating and stopping; iii) FT7 class is designated for athletes with unilateral spasticity, meaning that only one side of their body is affected, causing the players to walk and run with a limp. On the impaired side the athlete might have problems balancing, so that often the impaired leg is used to kick the ball; and iv) FT8 describes the minimum impairment criteria to be eligible and it is usually difficult to see the impact of impairment when watching the player running or controlling the ball. However, involuntary muscle contractions and hesitation before explosive movements do constitute activity limitations in comparison to regular football players.

Paralympic classification systems aim to promote participation in sport by people with disabilities by minimizing the impact of impairment on the outcome of competition [12], placing athletes in the same class when their impairments cause a similar degree of activity limitation [13]. Unfortunately, the system used for assessing and classifying brain impairments is typical of many Paralympic classification systems in which there is little scientific evidence on which to base methods for allocating these classes [14]. Evidence-based classification research is currently a hot topic in Paralympic sports and, in particular, evaluation of sprint ability has been recently applied in wheelchair propulsion in athletics [15, 16], runners with prosthetic legs [17], runners with brain injury [14], wheelchair rugby [18], or CP-Football [19]. The study by Reina et al. [19] demonstrated the validity and reliability of two COD tests to evaluate activity limitation in CP-Football players compared with controls. In fact, to the authors’ best knowledge, no previous studies have been focused on studying sprint and COD in para-footballers, comparing their performance with and without dribbling a ball.

Even though football requires accelerations, decelerations, and COD throughout the game, many sprints and COD tests are conducted around stationary objects. Whereas sprint running or pre-determined COD can be pre-planned (closed skill) [20], the inclusion of the ball during the test might increase its ecology [21]. For example, straight sprints in football are mostly conducted with the ball and it is the most frequent action in goal situations [22]. According to the new International Paralympic Committee Athlete Classification Code [23], International Sport Federations must develop sports-specific Classification Systems through multidisciplinary scientific research. Thus, it is necessary to evaluate general and specific football skills with standardized tests, in order to evaluate players´ activity limitations due to their impairments in a particular para-sport.

The aims of this study were: i) to analyze the absolute and relative intra-session reliability of three tests that require sprinting, accelerations, decelerations and COD (with and without ball) in para-footballers with brain impairments; ii) to evaluate the relationships among the tests used in the study; iii) to compare the performance in the tests when ball dribbling is required; and iv) to explore the practical implications for evidence-based classification in CP-Football and its usability for decision-making with the current classes. Hence, we hypothesized that the proposed tests to assess performance in CP-Football players will exhibit good intra-session reliability, being useful for decision-making between current class profiles. In addition, dribbling a ball during the tests will constrain para-footballers performance, requiring higher time to complete the tests.

## Materials and methods

### Participants

Eighty-two international para-footballers (n = 82, age = 25.2 ± 6.8 years; body mass = 68.7 ± 8.3 kg; height = 175.3 ± 7.4 cm; body mass index (BMI) = 22.5 ± 2.7 kg·m^-2^; competition experience = 10.8 ± 7.1 years) participated in the study. The participants were classified according to the IFCPF Classification Rules [11] (Table 1). At the moment of the data collection, 23.2% of the participants had taken part in the previous Paralympic Games, 66.3% in a worldwide competition, and 7.9% in a regional or continental competition. A group of 31 players without CP was also included in the study as a control group (CG) (Table 1). Inclusion criteria of the players for the CG were no impairment or injuries and having similar experience playing football with regard to para-footballers. Prior to involvement in the investigation, all participants gave written informed consent after a detailed written and oral explanation of the potential risks and benefits resulting from participation in this study, as outlined in the Declaration of Helsinki (2013). Approval by the institutional review board (Office for Projects Evaluation, OEP) was obtained before the study began (Ref. DPS.RRV.01.14). The participants had the option to voluntarily withdraw from the study at any time. Information about study aims, time commitment, risk and inconveniences, rights, potential benefits, responsibilities and ethical clearance was provided to all participants.

**Table 1:** Cerebral palsy football players’ characteristics.

Group	Impairment description	N	Age (years)	Body mass (Kg)	Height (cm)	BMI (Kg·m^-2^)	Training experience (years)
**FT5**	Bilateral spasticity (diplegia)	7	23.2 ± 6.4	70.0 ± 6.1	175.9 ± 6.1	21.7 ± 2.7	11.4 ± 5.2
**FT6**	Coordination impairments (dyskinesia or ataxia)	10	26.6 ± 8.7	65.3 ± 6.7	173.8 ± 5.3	21.8 ± 2.1	10.7 ± 3.3
**FT7**	Unilateral spasticity (hemiplegia)	51	24.9 ± 6.3	68.4 ± 8.2	175.2 ± 7.6	22.5 ± 2.9	10.0 ± 7.1
**FT8**	Minimum impairment criteria	14	26.5 ± 7.6	73.3 ± 7.9	176.7 ± 8.9	23.5 ± 2.2	13.6 ± 9.6
**CG**	No impairment	31	19.5 ± 3.3	72.4 ± 7.2	177.9 ± 5.7	22.9 ± 1.7	10.2 ± 5.1

BMI = body mass index; FT = Code reference for CP football classes, CG = control group.

### Procedures

All the para-footballers performed all tests on a football field during a CP-Football Worldwide Competition (i.e. 2013 CPISRA Football-7-a-side Intercontinental Cup, Barcelona, Spain). Tests were applied at least 24 hours after the last match. Before testing, a specific 10 min warm-up was performed by the participants, consisting of self-paced low-intensity run, skipping exercises, strides and two 15 m sprints with and without changes of direction. The order of administration of the tests was counterbalanced. Each participant performed every test twice, with and without the ball, and intra-session reliability was reported (3 tests x 2 conditions x 2 trials = 12 observations). A 3–5 min rest period was given between each trial [24]. Data collection on the CG was completed in two sessions at their training venue and at the same moment in the sport season. With players not previously familiarized, one familiarization trial was conducted to prevent a learning effect [25]. Every test was administered by the same tester for consistency.

#### Anthropometric measurements

The players’ heights were measured using a stadiometer with an accuracy of ± 1 mm (Harpenden, Holtain® Ltd., Crosswell, UK). Electronic scales (Oregon Scientific®, GR101, Portland, USA) with an accuracy of ± 0.01 kg were used to measure body mass. Body mass index was calculated by: (body mass in kg) / (height in m)^2^.

#### Illinois Agility Test (IAT)

**Fig 1:**
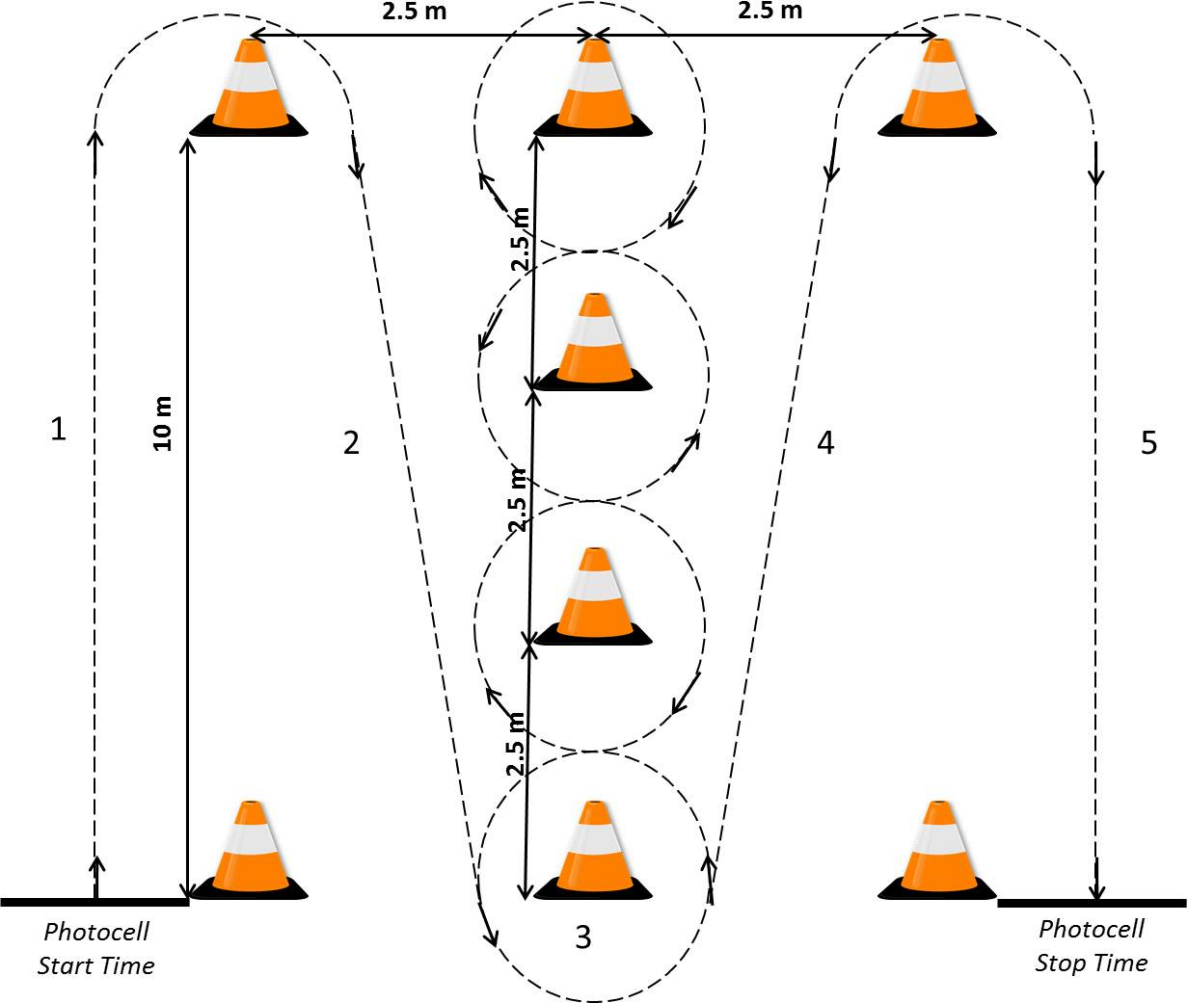
Schematic representation of Illinois Agility Test.

The IAT is set up with 4 cones forming the agility area (Fig 1), as used previously by Reina et al. [19] with CP-Football players, evaluating COD ability without (IAT) or with (IATB) ball dribbling. On command, (1) the athlete sprints 10 m, turns, and (2) returns to the starting line. After returning to the starting line, (3) he swerves in and out of 4 cones, (4, 5) completing two 10 m sprints to finish the agility course [26]. Performances were recorded using an electronic timing system (Globus®, Codogné, Italy) [19]. The infrared timing gates were positioned at the start and the finish line at a height of approximately 1 m.

#### Stop and Go Test (SGT)

**Fig 2:**
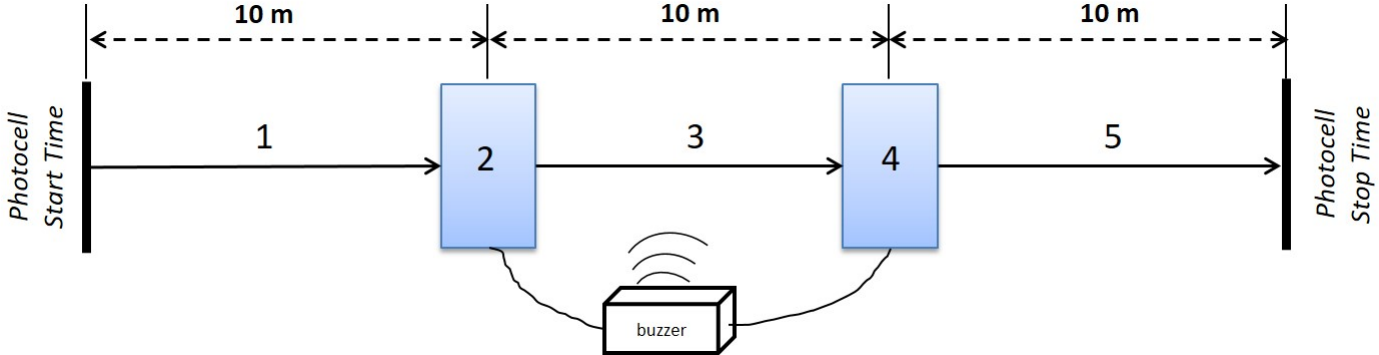
Schematic representation of Stop and Go test.

SGT is a new test applied in para-footballers and was designed from a test battery for evidence-based classification in CP-Football [27]. The test is set up with 2 contact mats and 2 infrared photocells (Fig 2). At his own discretion, (1) the athlete must run to the first 59 x 88 cm contact platform (Tapeswitch CVP-2335, Farmingdale, NY, USA), and (2) stop completely on the mat with both feet. (3) Two seconds after stepping up, a beep marks the next 10 m sprint to the second contact mat and (4) the athlete has to stop again on the mat. Finally, (5) two seconds after stepping up, a second beep sounds and athlete continues to the finish line. For the trials with the ball, the participants should control the ball on the mats until the beep sounds. The total time used to complete the test without (SGT) or with (SGTB) the ball was used for analysis. Performances were recorded using the same electronic timing system (Globus®, Codogné, Italy).

#### 40 m sprint (40S)

Sprinting performance was assessed using a sprint over a distance of 40 m, adapting the protocol used by Beckman and Tweedy [28]. The test was performed from a standing start and measured by means of infrared photocells (Globus®, Codogné, Italy) [19]. Participants were instructed to accelerate as quickly as possible through the timing gate positioned 40 m from the starting line. Time (s) was measured at 10 m (10S), 25 m (25S) and 40 m (40S) with and without ball (10SB, 25SB and 40SB).

### Statistical analysis

Results are presented as mean (M) ± standard deviation (SD). In each test, the two trials were used to analyze its reliability, and the best of the two trials to compare differences among conditions (with and without ball) and between groups. The Kolmogorov-Smirnov test was applied to evaluate the normal distribution of the collected data. All analyzed variables displayed a normal distribution; therefore, parametric statistics were used. Relative and absolute reliability between two trials in each test was assessed using intra-class correlations (ICC) and Standard Error of Measurement (SEM), respectively. The SEM was calculated using the following formula: *SEM* = SD * √1-ICC. Confidence interval limits for ICC and SEM were calculated at 90%. ICC values > 0.90 were considered excellent, 0.75–0.90 good and < 0.75 poor to moderate [29]. The coefficient of variation (CV, in %) was calculated within groups using the following formula: *CV* = (SD/Mean) * 100 [30]. The strength of association between the tests used in this study was assessed using a Pearson correlation (r). In order to interpret these results the threshold values for Pearson product-moment were used as presented by Salaj and Markovic [31]: low (r ≤ 0.3), moderate (0.3 < r ≤ 0.7) and high (r > 0.7). A repeated measures analysis of variance (ANOVA) was used, including the ball (yes/no) as a within-subject factor, and the CP-Football sub-classes and the CG as a between-group factor. Interactions between both factors were analyzed, and pair comparisons between groups were conducted with Tukey´s post-hoc analysis. Two effect size indexes were used to assess the practical significance within and between group differences. On the one hand, Partial eta-square (ηp2) values were calculated as a measure of effect size for mean differences with the following interpretation: above 0.26, between 0.26 and 0.02, and lower than 0.02 were considered as large, medium and small, respectively [32]. On the other hand, to calculate the effect size of post-hoc within-group differences, Hedges’ *g* index was used [33]. This index is based on Cohen’s *d* index [34], but it provides an effect size estimation reducing the bias caused by small samples (n < 20). Hedge’s *g* (*d*_g_) was interpreted according to Rhea´s proposal for professional or well-trained athletes [35]: above 1.00, between 0.50 and 1.00, between 0.25 and 0.50 and lower than 0.25 were considered large, moderate, small and trivial, respectively. Statistical significance was set at *P* < 0.05. Data analysis was performed using the Statistical Package for Social Sciences (SPSS Inc, version 23.0 for Windows, Chicago, IL, USA).

## Results

### Absolute and relative intra-session reliability

Para-footballers showed better reliability scores in their trials with and without ball, both for the ICC (IAT = 0.95, IATB = 0.84; SGT = 0.53, SGTB = 0.48; 10S = 0.71, 10SB = 0.84; 25S = 0.88, 25SB = 0.76; 40S = 0.85, 40SB = 0.73) and SEM (%) scores (IAT = 2.5, IATB = 6.3; SGT = 6.2, SGTB = 9.8; 10S = 4.6, 10SB = 4.5; 25S = 3.6, 25SB = 6.2; 40S = 3.9, 40SB = 6.5). In the case of CG, the ICC across tests was slightly lower (0.28–0.84) while SEM scored slightly higher (1.9–10.7%).

### Relationships between tests

Table 2 shows the correlations between tests and performance with and without ball.

**Table 2:** Pearson’s product moment correlation between the performance of the Illinois Agility Test, Stop and Go Test and Sprint Test with and without ball in para-footballers.

	IAT	IATB	SGT	SGTB	10S	10SB	25S	25SB	40S	40SB
**IAT**	—	0.28**	0.30**	0.37**	0.29*	0.34**	0.41**	0.34**	0.45**	0.47**
**IATB**			-0.08	0.35**	0.29*	0.55**	0.41**	0.50**	0.39**	0.34**
**SGT**				0.25*	0.23	0.29*	0.28*	0.35**	0.28*	0.36**
**SGTB**					0.40**	0.44**	0.41**	0.51**	0.30**	0.42**
**10S**						0.60**	0.82**	0.57**	0.81**	0.62**
**10SB**							0.70**	0.89**	0.66**	0.69**
**25S**								0.67**	0.85**	0.66**
**25SB**									0.59**	0.78**
**40S**										0.77**
**40SB**										—

IAT = Illinois Agility Test, IATB = Illinois Agility Test with ball, SGT = Stop and Go Test, SGTB = Stop and Go Test with ball, 10S = time at 10 m in Sprint test, 10SB = time at 10 m in Sprint test with ball 25S = time at 25 m in Sprint test, 25SB = time at 25 m in Sprint test with ball, 40S = 40 m Sprint test, 40SB = 40 m Sprint test with ball.

* p < 0.05.

** p < 0.01, significant correlation.

### Comparison between the tests with and without ball

The repeated measures ANOVA conducted using ball dribbling as a within-group factor showed significant differences in all the tests and variables in this study: IAT [F(4,95) = 262.54; p < 0.001; ηp2 = 0.73, large], SGT [F(4,95) = 61.56; p < 0.001; ηp2 = 0.39, large], 10S [F(4,95) = 62.58; p < 0.001; ηp2 = 0.44, large], 25S [F(4,95) = 30.29; p < 0.001; ηp2 = 0.69, large] and 40S [F(4,95) = 188.14; p < 0.001; ηp2 = 0.66, large]. Specifically, Table 3 shows significant differences and large effect sizes (0.53 < ηp2 < 0.97) when para-footballers and the CG performed the tests with and without dribbling the ball, except the FT8 in 10S and CG in SGT. In line with these results, players with moderate neurological impairments (i.e. FT5, FT6, and FT7) had higher coefficients of variation in the trial which requires ball dribbling: FT6 players show the higher CV in IAT and SGT, while in the straight sprint test, the CV are similar in FT5, FT6 and FT7 classes (with and without ball). Also, there are interaction effects among the within-group factor (ball dribbling) and the groups in all the tests: IAT [F(4,95) = 4.51; p = 0.002; ηp2 = 0.16, medium], SGT [F(4,95) = 7.28; p < 0.001; ηp2 = 0.24, medium], 40S [F(4,95) = 10.38; p < .001; ηp2 = 0.30, large]. The interaction effects were also obtained in the partial times in the sprint test: 10S [F(4,95) = 10.65; p < 0.001; ηp2 = 0.31, large] and 25S [F(4,95) = 9.04; p < 0.001; ηp2 = 0.28, large).

**Table 3:** Among group differences in all the tests performed with and without ball dribbling.

		Without Ball				With Ball							
		M		SD	CV	M		SD	CV	Ratio (%)	F	*p*	ηp2
**IAT** **(s)**	FT5	18.91	±	2.02	10.37	29.53	±	3.97	13.43	34.00	148.92	< 0.001	0.96
	FT6	20.10	±	2.75	13.81	29.60	±	5.96	20.15	32.77	17.62	0.002	0.64
	FT7	18.67	±	1.77	9.49	25.95	±	3.20	12.34	28.07	341.08	< 0.001	0.85
	FT8	17.75	±	1.24	7.01	22.61	±	2.42	10.47	23.30	73.12	< 0.001	0.84
	CG	15.91	±	0.67	4.21	21.18	±	0.88	4.13	24.89	980.54	< 0.001	0.97
**SGT** **(s)**	FT5	11.80	±	0.52	6.31	15.42	±	1.81	12.81	20.11	54.87	0.002	0.93
	FT6	13.71	±	2.59	16.52	15.58	±	1.80	11.25	36.60	280.48	< 0.001	0.97
	FT7	12.10	±	0.89	7.46	14.11	±	1.55	11.78	13.39	56.15	< 0.001	0.53
	FT8	11.75	±	1.15	8.70	13.73	±	1.62	13.87	16.99	24.43	< 0.001	0.69
	CG	11.61	±	0.52	4.45	11.86	±	1.51	13.64	2.16	0.86	0.360	0.03
**10S** **(s)**	FT5	2.17	±	0.10	4.51	2.55	±	0.32	12.38	14.85	5.86	0.019	0.66
	FT6	2.03	±	0.16	7.73	2.34	±	0.20	8.61	13.29	16.95	0.004	0.71
	FT7	2.02	±	0.14	6.84	2.18	±	0.18	8.09	7.31	62.93	< 0.001	0.58
	FT8	1.91	±	0.15	7.73	1.95	±	0.13	6.74	1.85	0.27	0.616	0.03
	CG	1.93	±	0.14	7.49	1.96	±	0.15	7.51	1.83	6.19	0.019	0.18
**25S** **(s)**	FT5	2.37	±	0.25	10.59	2.96	±	0.32	10.82	19.80	43.62	0.007	0.94
	FT6	2.27	±	0.19	8.30	2.84	±	0.29	10.17	19.98	32.03	0.001	0.82
	FT7	2.20	±	0.22	9.84	2.61	±	0.27	10.25	15.80	193.03	< 0.001	0.81
	FT8	2.01	±	0.16	8.01	2.32	±	0.19	8.10	13.67	41.54	< 0.001	0.82
	CG	1.90	±	0.17	9.12	2.10	±	0.13	6.32	9.38	34.58	< 0.001	0.54
**40S** **(s)**	FT5	6.88	±	0.56	8.11	8.00	±	0.82	10.24	14.00	41.62	0.008	0.93
	FT6	6.58	±	0.56	8.44	8.05	±	0.85	10.53	18.25	36.29	0.001	0.84
	FT7	6.43	±	0.56	8.78	7.44	±	0.77	10.41	13.56	195.21	0.001	0.81
	FT8	5.90	±	0.54	9.13	6.66	±	0.51	7.73	11.48	28.57	< 0.001	0.76
	CG	5.68	±	0.32	5.61	6.09	±	0.35	5.78	6.71	60.18	< 0.001	0.68

M = mean, SD = standard deviation, CV = coefficient of variation, IAT = Illinois Agility Test, SGT = Stop and Go Test, 10S = time at 10 m in Sprint test, 25S = time at 25 m in Sprint test, 40S = 40m Sprint test, FT = CP-football class, CG = control group.

### Between-groups comparisons

For all the tests, and the partial times in the sprint test, we also obtained between-group statistical and large practical differences: IAT [F(4,95) = 39.22; p < 0.001; ηp2 = 0.62, large], SGT [F(4,95) = 16.93; p < 0.001; ηp2 = 0.42, large], 40S [F(4,95) = 27.89; p < .001; ηp2 = 0.54, large], 10S [F(4,95) = 12.79; p < 0.001; ηp2 = 0.35, large] and 25S [F(4,95) = 30.88; p < 0.001; ηp2 = 0.57, large). Table 4 shows the pair comparisons between groups. In the IAT, the CG shows differences with regards all the CP-Football profiles (0.92 < *d*_g_ < 4.52, moderate-to-large), while the FT8 para-footballers only showed differences with the other three classes in the test performed with ball. In the SGT without ball, FT6 is the group of para-footballers with greater differences with regard to the others, and the CG had differences with all the CP-Football classes in the test with ball (1.19 < *d*_g_ < 2.31, large). In the sprint tests (10, 25 and 40 m), the differences between CG and para-footballers were more evident in the test with ball (1.29 < *d*_g_ < 4.77, large), although no difference was obtained regarding the FT8 group in the first 10 m (acceleration phase). In the sprint without ball, the differences between groups were more evident in the longer distance (i.e. 40 m).

**Table 4:** Between-group differences (Tukey´s post hoc) and effect sizes (*d*_g_) in all the tests performed with and without ball dribbling.

		Tests without ball					Tests with ball				
		FT5	FT6	FT7	FT8	CG	FT5	FT6	FT7	FT8	CG
**IAT**	FT5	—	-0.45	0.13	0.73	2.86**	—	-0.01	1.07*	2.21**	4.52**
	FT6	0.45	—	0.72	1.13**	2.84**	0.01	—	0.96**	1.59**	2.78**
	FT7	-0.13	-0.72	—	0.54	1.87**	-1.07**	-0.96**	—	1.08*	1.82**
	FT8	-0.73	-1.13	-0.54	—	2.05**	-2.21**	-1.59**	-1.08*	—	0.92
	CG	-2.86**	-2.84**	-1.87**	-2.05**	—	-4.52**	-2.78**	-1.82**	-0.92*	—
**SGT**	FT5	—	-0.89	-0.34	0.05	0.36	—	-0.08	0.82	0.96	2.23**
	FT6	0.89	—	1.22**	1.01*	1.55**	0.08	—	0.91	1.05	2.31**
	FT7	0.34	-1.22**	—	0.36	0.63	-0.82	-0.91	—	0.24	1.45**
	FT8	-0.05	-1.01*	-0.36	—	0.18	-0.96	-1.05	-0.24	—	1.19**
	CG	-0.36	-1.55**	-0.63	-0.18	—	-2.23**	-2.31**	-1.45**	-1.19**	—
**10S**	FT5	—	0.96	1.09	1.83**	1.75**	—	0.78	1.82**	2.75**	3.05**
	FT6	-0.96	—	0.07	0.75	0.68	-0.78	—	0.86*	2.32**	2.29**
	FT7	-1.09	-0.07	—	0.76	0.64*	-1.82**	-0.86*	—	1.33**	1.29**
	FT8	-1.83**	-0.75	-0.76	—	-0.14	-2.75**	-2.32**	-1.33**	—	-0.07
	CG	-1.75**	-0.68	-0.64*	0.14	—	-3.05**	-2.29**	-1.29**	0.07	—
**25S**	FT5	—	0.44	0.75	1.79**	2.48**	—	0.38	1.25*	2.57**	4.77**
	FT6	-0.44	—	0.32	1.45*	2.08**	-0.38	—	0.83*	2.13**	4.03**
	FT7	-0.75	-0.32	—	0.90*	1.47**	-1.25*	-0.83*	—	1.12**	2.22**
	FT8	-1.79**	-1.45*	-0.90	—	0.65	-2.57**	-2.13**	-1.12**	—	1.43*
	CG	-2.48**	-2.08**	-1.47**	-0.65	—	-4.77**	-4.03**	-2.22**	-1.43*	—
**40S**	FT5	—	0.51	0.79	1.72**	3.17**	—	-0.06	0.71	2.06**	4.04**
	FT6	-0.51	—	0.26	1.20*	2.27**	0.06	—	0.77	2.00**	3.76**
	FT7	-0.79	-0.26	—	0.94*	1.53**	-0.71	-0.77	—	1.06**	2.07**
	FT8	-1.72**	-1.20*	-0.94*	—	0.54	-2.06**	-2.00**	-1.06**	—	1.38*
	CG	-3.17**	-2.27**	-1.53**	-0.54	—	-4.04**	-3.76**	-2.07**	-1.38*	—

IAT = Illinois Agility Test, SGT = Stop and Go Test, 10S = time at 10 m in Sprint test, 25S = time at 25 m in Sprint test, 40S = 40m Sprint test, FT = CP-football class, CG = control group.

** p < 0.01.

* p < 0.05.

## Discussion

Paralympic classification systems must initially determine eligibility, based on criteria which describe the types of impairment that are eligible in a particular sport and how severe they must be, comprising methods for assessing and classifying eligible impairments according to the extent of activity limitation they cause [12]. This study reports good absolute and relative intra-session reliability of a battery of tests to assess sprint, accelerations/decelerations and COD in para-footballers with neurological impairments, finding relationships among tests that evaluate different skills required for CP-Football proficiency. As we hypothesized, the presence of the ball during tests execution constraints athletes´ performance (i.e. more time is required), and the test battery also allow discriminating between CP-Football classes (FT5/6/7 v FT8), and also between para-footballers and controls.

Previous studies have analyzed sprinting [13] and COD [19] in athletes with neurological impairments. To the authors´ knowledge, no previous studies have analyzed performance including ball dribbling during testing, so the feasibility and reliability of those measurements have not been studied yet. Relative (ICC) and absolute (SEM) reliability were analyzed in para-footballers and controls in this study, indicating acceptable reliability in para-footballers. Reproducibility in a test that involved ball dribbling is challenging, and the inclusion of the ball transforms the test from a closed to a more open task [20], constraining the para-footballers performance (i.e. decreasing ICC and increasing SEM values). Having found moderate-to-excellent reliability scores in para-footballers, it is plausible to think that athletes with neurological impairments affecting lower limbs (i.e. bilateral/unilateral spasticity or coordination impairments) may have more difficulties to control the ball when they are performing a maximum trial, being less adaptable to mistakes during dribbling. Nevertheless, the study by Mirkov et al. [36] with professional football players did report lower coefficient of variation, higher intra-class correlation coefficient and lower typical error of measurement in zig-zag agility tests without and with ball respectively. This hypothesis can be also supported with the low-to-moderate correlations obtained between tests with and without ball, specifically in the IAT (r = .275), SGT (r = .697) and 40S (r = .249) in para-footballers; and IAT (r = .651) and SGT (r = .697) in controls. So, the proposed test may be applicable for classification purposes, that is, evaluation of activity limitation from neurological impairments or decision-making between current CP-Football classes.

The low-to-moderate or no correlations obtained between the tests used in this study are in line with other studies in the literature on this topic [31, 37, 38], suggesting that they are measuring distinct qualities [6, 39], with different tests durations [40]. COD involves fast accelerations that convert into sudden decelerations requiring high–eccentric strength gradients [5, 6], including different angles for COD [9, 40]. However, this is not required in straight sprints [41]. In our study, IAT required smooth and sudden COD, while SGT required sudden accelerations and decelerations, and these tests are normally used in classification to evaluate the impact of the eligible impairments in CP-Football. In fact, it has been demonstrated that people with cerebral palsy often have difficulties changing direction of the body abruptly or shifting quickly the direction of movement without losing balance [42]. Considering the moderate correlations between tests, and the differences among trials with and without ball dribbling, it would be reasonable to think that these tests measure different capabilities in CP-football, making its inclusion feasible in classification processes in order to evaluate different aspects from the different eligible impairments and profiles.

Regarding the effect of the ball during testing, there are not many studies in the literature that compare sprint and COD ability with and without ball [39], and this study is the first one to analyze them in para-footballers. According to Köklü et al. [39], agility with and without ball tests are affected by different factors, including the technical capacity of players, to evaluate different soccer skills. Our results are supported with higher CV found, in general, in the tests with ball, and a longer time recorded in all the tests performed with ball. However, the tests with ball may not be considered a true open skill task because other players are not involved. These results agree with those of Köklü et al. [39], who did not find correlations between a zig-zag agility test with the ball and vertical jump performance, acceleration, and maximum speed in young soccer players, arguing that players’ technical skill levels become a factor in zig-zag agility performance with the ball. COD ability has been reported to be course specific and as a “per se” physical ability [31, 37], and due to the demonstrated specificity in training effect, the need for sport and tactic-specific improvement in COD abilities is warranted [43]. The ratio of difference among tests with and without ball is usually higher in players from classes FT5 and FT6. For example, in IAT, FT5 players may demonstrate lower ability in displacements and COD because of bilateral spasticity in the lower limbs, while FT6 players may require a lot of adjustments during the test pathway due to their typical coordination problems from ataxia or athetosis. These differences can also be observed in straight sprint times, demonstrating their problems in starting, accelerating and running at top speed. In addition, FT8 players also showed a higher ratio of differences than controls, demonstrating that the proposed tests, with and without ball, can be valid measures to evaluate activity limitation in para-footballers with neurological impairments.

Valid systems of classification should ensure that the successful athletes will be those who have the most advantageous combination of anthropometric, physiological, and/or psychological attributes, and have enhanced them to the best effect [12]. For instance, athletes would not succeed simply because their impairments are less severe than those of their competitors [12]. CP-Football shows the so-called cut-point problem [27], where classifiers usually decide between moderate-to-mild spastic diplegia (FT5-FT8), moderate-to-mild athetosis or ataxia (FT6-FT8) and moderate-to-mild spastic hemiplegia (FT7-FT8). The ambiguity of some parameters included in the profiles definition is a threat to the validity of the CP-Football classification system. This fact is demonstrated by the reduction from 2 FT8 players to only 1 allowed in the squad during the game after the 2012 London Paralympic Games, and the general opinion about current FT8 players in CP-Football that they should not play because of their significantly higher (or non-visibly affected) football skills [44]. Similarly, after the 2016 Rio Paralympic Games, IFCPF decided to increase the number of players from classes FT5 or FT6 (from 1 to 2) that must play during the game [45]. The differences between groups (Para-footballers vs. CG, and among CP-Football classes) demonstrated the activity limitation provoked by the eligible impairments for this para-sport, except some comparisons such as FT8 in the time at S10. Boyd et al. [46] investigated the football match-play work of players with CP, demonstrating that FT8 players had the higher maximum speed of high intensity and very high-intensity activity compared with the other CP-Football profiles, but no differences were observed between FT5/6 and FT7 classes. They concluded that FT8 players display, most notably, a better performance in very high-intensity activity associated with game-defining moments, while FT5/6 and FT7 players performed equitably. Considering this cut-point problem [27], FT5, FT6 and FT7 have been usually considered as different profiles of “moderate” impairments, but our results showed significant differences and/or moderate to large effect sizes between FT7 and FT5/6 para-footballers in all the tests, both with and without ball dribbling. For example, Reina et al. [19] demonstrated that two distinct COD tests (MAT and IAT) can be used differently to check the activity limitation in players with spastic diplegia (FT5), spastic hemiplegia (FT7) and players with involuntary movements or impaired movement control (FT6). Thus, the test battery proposed here may also help classifiers in their decision-making to evaluate among different profiles.

Beckman et al. [13] found significant differences between strength and athletic performance values (decreased sprint scores) in runners with brain injury when compared with controls. However, their results showed that in athletes with mild impairments strength is not an important predictor of running performance, suggesting the need to evaluate the relationship between strength and performance in athletes with more severe impairments to muscle strength for determining whether strength is a limiting factor in running performance. In fact, our results showed that players in the classes FT5, FT6 and FT7 had lower sprint performance (10, 25, 40 m) compared with FT8 players and controls. In addition, the differences between the two CP-Football sub-groups with lower limbs spasticity (FT5, bilateral; FT7, unilateral) was found in the 10 m distance, when a sudden acceleration is required. Upper motor lesions such as CP cause atrophy of type II (fast) muscle fibers, resulting in a greater proportion of type I (slow) muscle fibers [47], and spasticity may be the result of intrinsic modifications of the muscle and/or altered reflex properties [48]. Muscle strength and anaerobic power of the lower extremities are neuromuscular variables that influence performance in many sports activities, including football [49], and they have been reported to be distinctly weaker in individuals with CP [50, 51]. A recent study demonstrated that young football players who have good hamstring flexibility obtained better performance scores in tests of acceleration and sprinting, considering it to be a key factor for performing football-specific skills, such as sprinting, jumping, agility, and kicking [52]. In addition, Jung, Her, and Ko [53] indicated that the plantar flexors may be the muscle group which has the greatest impact on activity limitation, as it has been shown to be the weakest in the lower limbs in children with CP when compared with typically developing children.

Besides the study in para-athletic sprinting by Beckman et al. [13], activity limitation has been demonstrated in running [54], sprinting and COD [19, 42]. The study by Verschuren et al. [42] included a 10 x 5 m sprint test, that is, an intermittent sprint test where the participant stops and starts at standardized intervals, finding large standard deviations because of inter-individual variability. This variability is particularly typical in FT6 players, characterized by coordination problems from ataxia or athetosis. Besides the differences between FT8 and the CG, this sub-group obtained significant differences regarding FT7 players in the SGT. It is plausible that the players with spastic hemiplegia (FT7) use their less affected side for sudden accelerations-decelerations and ball dribbling. Considering the minimum of players from the classes FT5/6 and the maximum from the class FT8 [45], there are no special considerations about the number of players from class FT7 during the game, so at least 4 of the 7 players usually belong to this class. A player with frequent contact with the ball is involved in more game situations, which may lead to increased performance [55]. Involvements with the ball are also influenced by anthropometric characteristics [56], and FT7 players have a non-affected leg to perform typical football skills. Thus, playing position is another factor to be considered in football because of different physical activity requirements during a match, also demonstrated in the repeated sprint test [57]. Some studies (i.e. [58]) have consistently shown that defenders cover less distance with high-intensity running and sprinting compared to the other playing positions. In CP-Football, FT5 players usually play as goalkeeper because of their limited stride, jumping or kicking skills (limited follow through because of lower limbs spasticity), and also due to their minimal or no impairment in the upper limbs. However, FT6 players usually play as defenders or midfielders, minimizing the impact of their coordination problems. The lowers scores obtained by these two groups in all the tests included in this study evidence their higher activity limitation.

Some limitations should be mentioned. The development of evidence-based classification systems in Paralympic sports, whenever is possible, require resistant to training measurements [12]. With a view to developing evidence-based classification systems, current best practice requires classification panels to assign a class by collectively considering outcomes from the impairment assessment (hypertonia, ataxia or athetosis), together with three other forms of assessment [59]: i) novel motor tasks, which are tasks that are unlikely to have been practiced by the athlete in the usual course of training for his/her sport; ii) sport-specific activities that are likely to have been frequently practiced by athletes training for a particular para-sport; and iii) a detailed training history and other personal and environmental factors likely to affect sport proficiency. Although a novel task may be more resistant to training, it might be difficult to found valid and reliable sport-specific field test resistant to training in para-footballers, because several skills determine proficiency in this para-sport. However, the participants of this study may be considered well-trained athletes (i.e. data collection was conducted in a world competition), and they were selected as the best athletes from their countries, but their level of training was not considered. Future studies are required to address this limitation, conducting longitudinal or between-sessions reliability studies to evaluate the training/practice effect of testing.

## Conclusions and practical implications

This study is the first one to evaluate straight sprinting, sudden accelerations-decelerations and COD with and without ball, both in football and para-football for players with brain impairments. The tests performed are useful for evaluating activity limitation in para-footballers with neurological impairments, allowing discrimination among FT8 players (i.e. minimal impairment criteria) and controls. The relationships among tests also evidence that they measure different capabilities, and the inclusion of the ball influences testing reproducibility.

The IAT was previously applied in para-footballers [19], involving acceleration, as well as directional changes when sprinting in a linear fashion. Although the IAT can last for approximately 15–19 s (21–29 s with ball), it might be considered by classifiers a good test for observing activity limitation in the required skills. With regard to the SGT, it appears to be a standardized test to evaluate both novel and sport-specific skills in CP-Football. However, the use of the contact mat to activate the sensor is a variable to consider in future studies, because the different surface might slide when it is placed on natural turf, impacting on the players “feeling” to accelerate (start) and decelerate (stop) wearing football boots. On the other hand, the 40m distance was included because of the different proposals to evaluate sprint in football, and it is the only one study applied in para-athlete runners [28]. However, Stølen et al. [1] demonstrated that during a game 96% of sprints are less than 30 m, and it is not enough to reach the maximal individual speed [60].

With a view to developing an evidence-based classification system for CP-Football, the tests performed in this study have been included in the classification protocols by IFCPF, and further research is necessary to develop sport-specific tests for this population that combine different football skills such as straight sprinting and/or COD (i.e. CODAT) [61].

## Supporting information

S1 Dataset Spreadsheet Excel file. (XLSX)
